# SmgGDS: An Emerging Master Regulator of Prenylation and Trafficking by Small GTPases in the Ras and Rho Families

**DOI:** 10.3389/fmolb.2021.685135

**Published:** 2021-06-16

**Authors:** Anthony C. Brandt, Olivia J. Koehn, Carol L. Williams

**Affiliations:** Department of Pharmacology and Toxicology, Medical College of Wisconsin, Milwaukee, WI, United States

**Keywords:** Rap1GDS1, SmgGDS, splicing, lipidation, prenylation, trafficking, GEF, GDF

## Abstract

Newly synthesized small GTPases in the Ras and Rho families are prenylated by cytosolic prenyltransferases and then escorted by chaperones to membranes, the nucleus, and other sites where the GTPases participate in a variety of signaling cascades. Understanding how prenylation and trafficking are regulated will help define new therapeutic strategies for cancer and other disorders involving abnormal signaling by these small GTPases. A growing body of evidence indicates that splice variants of SmgGDS (gene name RAP1GDS1) are major regulators of the prenylation, post-prenylation processing, and trafficking of Ras and Rho family members. SmgGDS-607 binds pre-prenylated small GTPases, while SmgGDS-558 binds prenylated small GTPases. This review discusses the history of SmgGDS research and explains our current understanding of how SmgGDS splice variants regulate the prenylation and trafficking of small GTPases. We discuss recent evidence that mutant forms of RabL3 and Rab22a control the release of small GTPases from SmgGDS, and review the inhibitory actions of DiRas1, which competitively blocks the binding of other small GTPases to SmgGDS. We conclude with a discussion of current strategies for therapeutic targeting of SmgGDS in cancer involving splice-switching oligonucleotides and peptide inhibitors.

## Introduction

The Ras superfamily consists of over 150 different small GTPases belonging to specific families. The most well characterized small GTPases in this superfamily are members of the Ras family (36 members), Rho family (20 members), and Rab family (over 60 members) (Vigil et al., 2010; Gray et al., 2020). These proteins participate in important cellular signaling pathways that regulate gene expression, cytoskeletal organization, intracellular trafficking of proteins and vesicles, and cell migration, proliferation, and differentiation (Seabra et al., 2002; Hutagalung and Novick, 2011; Haga and Ridley, 2016) These small GTPases are activated when they bind guanine nucleotide exchange factors (GEFs) that induce the small GTPases to release GDP and bind GTP. There are 27 different GEFs that activate Ras family members, and 80 GEFs that activate Rho family members, providing extensive spatiotemporal control of these small GTPases (Vigil et al., 2010; Gray et al., 2020). Inappropriate or prolonged activation of these GTPases leads to dysregulated signaling that contributes to cancer initiation, progression, and metastasis (Seabra et al., 2002; Vigil et al., 2010; Alan and Lundquist, 2013; Haga and Ridley, 2016; Porter et al., 2016).

The intracellular site where a small GTPase is located defines how the GTPase will be activated and which signaling pathway it will modulate. Cell membranes are a major site for activation and signaling by small GTPases. Ras and Rho family members anchored at the plasma membrane are activated by membrane-localized GEFs and participate in signaling cascades initiated by plasma membrane receptors (Vigil et al., 2010; Gray et al., 2020). Rab family members anchored at endosomal membranes are activated by endosome-localized GEFs and participate in vesicular transport (Seabra et al., 2002; Hutagalung and Novick, 2011). The ability of these GTPases to anchor at membranes and participate in these signaling events depends on the presence of a prenyl group that is irreversibly attached to the C-terminus of the GTPase soon after it is synthesized in the cell (Lane and Beese, 2006; Wright and Philips, 2006; Wang and Casey, 2016). The prenyl group serves as a membrane anchor that is inserted into the lipid bilayer. If prenylation does not occur, the ability of these GTPases to localize at cell membranes is severely impaired.

Small GTPases that are activated by GEFs associated with membranes must be prenylated to localize at the membrane and interact with their GEFs. K-Ras4B is an excellent example of a small GTPase that relies primarily on membrane localization for its activity (Cox et al., 2015; Kattan and Hancock, 2020; Uprety and Adjei, 2020). However, recent studies indicate that under certain conditions, K-Ras4B might participate in signaling complexes that are not associated with membranes (Tulpule et al., 2021). Some small GTPases are known to be activated at sites other than membranes. For example, the Ras family members Rap1A and Rap1B (Mitra et al., 2003; Goto et al., 2010; Ntantie et al., 2013; Griffin et al., 2018) and the Rho family members RhoA (Dubash et al., 2011; Staus et al., 2014; Gayle et al., 2015) and Rac1 (Lanning et al., 2003; Michaelson et al., 2008; Huff et al., 2013; Navarro-Lerida et al., 2015; Justilien et al., 2017; Casado-Medrano et al., 2020) can enter the nucleus where they can be activated by nuclear GEFs and participate in nuclear signaling pathways. These findings suggest that prenylation that promotes membrane anchoring is not always required for Ras and Rho family members to become activated. This suggestion is supported by reports that inhibiting the prenylation of Rap1A, Rap1B, RhoA, and Rac1 increases the GTP-bound forms of these GTPases (Dunford et al., 2006; Khan et al., 2011; Khan et al., 2013; Ntantie et al., 2013; Reddy et al., 2015; Akula et al., 2019), indicating that certain GEFs can activate these GTPases before they are prenylated.

There is growing evidence that some small GTPases participate in signaling events before they are prenylated (Khan et al., 2011; Khan et al., 2013; Reddy et al., 2015; Akula et al., 2019), leading to the realization that cells must possess ways to promote or suppress the prenylation of a newly synthesized small GTPase. The best characterized mechanism that controls the prenylation of Ras and Rho family members involves the interaction of these small GTPases with SmgGDS (pronounced “smidge-G-D-S”, gene name RAP1GDS1). SmgGDS has emerged as a major regulator of both the prenylation and intracellular trafficking of many GTPases in the Ras and Rho families (Berg et al., 2010; Ntantie et al., 2013; Williams, 2013; Schuld N. J. et al., 2014; Jennings et al., 2018; Garcia-Torres and Fierke, 2019; Nissim et al., 2019; Brandt et al., 2020; Liao et al., 2020). This review describes how these events are regulated by the two splice variants of SmgGDS, named SmgGDS-607 and SmgGDS-558, and compares SmgGDS to the proteins that regulate the prenylation and trafficking of Rab family members. We discuss how different proteins modulate the interactions of SmgGDS with oncogenic small GTPases in the Ras and Rho families, and present strategies to target SmgGDS therapeutically in cancer.

## Prenylation of Ras, Rho, and Rab Family Members

Newly synthesized small GTPases in the Ras and Rho families are soluble, hydrophilic proteins residing in the cytosol. The majority of these small GTPase have a C-terminal CAAX motif consisting of a cysteine (C), two aliphatic amino acids (AA), and a terminal amino acid (X). When a newly synthesized small GTPase enters the prenylation pathway, the CAAX motif undergoes prenylation and post-prenylation processing, converting the small GTPase to a hydrophobic protein that can anchor at membranes (Lane and Beese, 2006; Wright and Philips, 2006; Wang and Casey, 2016).

Ras and Rho family members are prenylated by a cytosolic prenyltransferase (PTase), which irreversibly attaches a hydrophobic prenyl group to the cysteine in the CAAX motif. Small GTPases that have a CAAX motif ending in alanine, glycine, serine, methionine, or phenylalanine receive a 15-carbon farnesyl group from farnesyltransferase (FTase). In contrast, if the CAAX motif ends in leucine or phenylalanine, the GTPase receives a 20-carbon geranylgeranyl group from geranylgeranyltransferase-I (GGTase-I) (Lane and Beese, 2006). The prenylated GTPase then undergoes post-prenylation processing at the endoplasmic reticulum by interacting with RCE1, which proteolytically cleaves the AAX from the CAAX motif, followed by carboxylmethylation by ICMT (Wright and Philips, 2006; Wang and Casey, 2016).

When post-prenylation processing is completed, the prenylated, hydrophobic GTPase can take two different routes to the plasma membrane. Small GTPases that have an additional cysteine near the CAAX motif, such as H-Ras and N-Ras, move to the Golgi to become palmitoylated before localizing at the plasma membrane (Wright and Philips, 2006; Wang and Casey, 2016). In contrast, small GTPases that have a C-terminal polybasic region (PBR) move directly from the endoplasmic reticulum through the aqueous cytosol to the plasma membrane (Wright and Philips, 2006; Wang and Casey, 2016). These PBR-containing small GTPases include K-Ras4B and many other members of the Ras and Rho families [reviewed in Williams (2013)] (Table 1). A protein that serves as a chaperone must shield the prenyl group of the small GTPase in a hydrophobic pocket as the GTPase moves through the cytosol to the plasma membrane (Azoulay-Alfaguter et al., 2015). Prenylated Ras family members interact with several chaperones, including PDEδ (Bhagatji et al., 2010; Dharmaiah et al., 2016), PRA1 (Figueroa et al., 2001; Bhagatji et al., 2010), and VPS35 (Zhou et al., 2016), while prenylated Rho family members are chaperoned by three RhoGDI proteins (Garcia-Mata et al., 2011).

**TABLE 1:** C-Terminal Sequences of Human Ras and Rho Family Members that have a Polybasic Region (PBR).

GTPase	C-terminal sequence^a^	Accession No.^b^
Rho family members		
RhoA	fematRaalqaRRgKKKsgclvl	NP_001300870
RhoC	fematRaglqvRKnKRRRgcpil	NP_001036144
RhoD	evalssRgRnfwRRitqgfcvvt	NP_055393
RhoG	faeavRavlnptpiKRgRscill	NP_001656
RhoH	tavnqaRRRnRRRlfsineckif	NP_001265298
Rac1	deaiRavlcpppvKKRKRKclll	NP_008839
Rac1b	deaiRavlcpppvKKRKRKclll	NP_061485
Rac2	deaiRavlcpqptRqqKRacsll	NP_002863
Rac3	deaiRavlcpppvKKpgKKctvf	NP_005043
Cdc42 (isof. 1)	fdeailaaleppepKKsRRcvll	NP_001782
Cdc42 (isof. 2)	fdeailaaleppetqpKrKccif	NP_426359
Rnd1 (Rho6)	lpsRselisstfKKeKaKscsim	NP_055285
Rnd2 (RhoN)	sgrpdRgnegeihKdRaKscnlm	NP_005431
Rnd3 (RhoE)	sRpelsavatdlRKdKaKsctvm	NP_005159
Ras family members		
K-Ras4A	RlKKisKeeKtpgcvKiKKciim	NP_001356715
K-Ras4B	hKeKmsKdgKKKKKKsKtKcvim	NP_001356716
R-Ras2 (TC21)	ecppspeptRKeKdKKgchcvif	NP_036382
M-Ras (R-Ras3)	KKKKtKwrgdRatgthKlqcvil	NP_001239019
Rap1A	vRqinRKtpveKKKpKKKsclll	NP_001010935
Rap1B	vRqinRKtpvpgKaRKKsscqll	NP_001010942
RalA	KeKngKKKRKslaKRiReRccil	NP_005393
RalB	nKdKngKKssKnKKsfKeRccll	NP_001356329
Rheb	RiileaeKmdgaasqgKsscsvm	NP_005605
RhebL1	tKviqeiaRvensygqeRRchlm	NP_653194
DiRas1 (Rig)	nidgKRsgKqKrtdRvKgKctlm	NP_660156
DiRas2	qidgKKsKqqKRKeKlKgKcvim	NP_060064
DiRas3	qepeKKsqmpntteKlldKciim	NP_004666

a Basic amino acids that make up the PBR are capitalized.

b The NCBI Protein database accession number is provided.

Rab family members are prenylated in a pathway differing from the one that prenylates Ras and Rho family members. Newly synthesized Rab family members associate with the Rab Escort Protein REP1 before prenylation. A trimeric complex consisting of REP1, the Rab protein, and the Rab geranylgeranyltransferase (RabGGTase) is needed for the RabGGTase to sequentially prenylate two cysteines in the C-terminus of the Rab protein. After prenylation, REP1 serves as a chaperone for the prenylated Rab small GTPase as it moves through the cytosol to membranes. The importance of REP1 in this pathway is indicated by its interactions with both the pre-prenylated and prenylated forms of the Rab protein, facilitating prenylation of the newly synthesized Rab protein and then escorting the prenylated Rab protein to membranes (Preising and Ayuso, 2004; Goody et al., 2005; Leung et al., 2006; Wu et al., 2007).

The participation of REP1 in the prenylation and trafficking of newly synthesized Rab family members suggests that proteins with functions similar to REP1 might participate in the prenylation and trafficking of newly synthesized Ras and Rho family members. Such proteins that might assist Ras and Rho family members during prenylation were not known prior to the discovery of the two major splice variants of SmgGDS (Berg et al., 2010). The discovery of these SmgGDS splice variants has led to an increasing understanding of how cells can suppress or promote the prenylation of Ras and Rho family members, and has stimulated a growing exploration of how Ras and Rho family members can actively signal both before and after they are prenylated.

## Discovery of SmgGDS and its Major Splice Variants SmgGDS-607 and SmgGDS-558

In 1990, members of the Takai laboratory isolated a protein from bovine brain that interacted with Rap1A and Rap1B, and they named this protein “small G protein guanine dissociation stimulator” or SmgGDS (Yamamoto et al., 1990). A cDNA encoding a SmgGDS protein having 558 amino acids was generated (Kaibuchi et al., 1991), and this SmgGDS cDNA was utilized in many studies to define the functions of SmgGDS. These studies indicated that SmgGDS binds multiple members of the Ras and Rho families that have a C-terminal PBR, including K-Ras4B, Rap1A, Rap1B, RhoA, RhoC, Rac1, Rac2, and Cdc42 (Mizuno et al., 1991; Hiraoka et al., 1992; Kikuchi et al., 1992; Orita et al., 1993; Yaku et al., 1994; Xu et al., 1997). Co-expression of this 558 amino acid form of SmgGDS with different small GTPases enhanced several cellular responses, including transformation and tumorigenesis of NIH3T3 cells induced by K-Ras4B (Fujioka et al., 1992), lamellipodia formation promoted by Rap1B (Yoshida et al., 1992), and NOX activation and neurite formation induced by Rac1 (Ando et al., 1992; Kikuchi et al., 1992; Mizuno et al., 1992).

SmgGDS has been the subject of several controversies regarding its interactions with small GTPases. One of these controversies arose from inconsistent reports that a small GTPase must be prenylated before it can associate with SmgGDS. Some groups reported that SmgGDS interacts only with prenylated small GTPases (Mizuno et al., 1991; Shirataki et al., 1991; Fujioka et al., 1992; Orita et al., 1993; Nakanishi et al., 1994), whereas others reported that SmgGDS can associate with small GTPases before they are prenylated (Chuang et al., 1994; Hutchinson and Eccleston, 2000; Hutchinson et al., 2000). This discrepancy might have occurred because these groups used different cDNAs encoding SmgGDS in their studies. Groups reporting that SmgGDS binds only prenylated GTPases utilized the cDNA that was generated in the original studies of SmgGDS (Mizuno et al., 1991; Nakanishi et al., 1994). In contrast, groups reporting that prenylation was unnecessary utilized SmgGDS cDNA clones generated in other studies (Chuang et al., 1994; Hutchinson and Eccleston, 2000; Hutchinson et al., 2000). The use of these different cDNA clones was not considered to be an important variable at the time, but it might have explained the disparate results obtained in these studies, if the cDNAs being utilized by these different groups encoded different forms of SmgGDS.

An explanation for these conflicting reports that only prenylated GTPases bind SmgGDS was provided in 2010, when the Williams group reported the identification of two splice variants of SmgGDS that differ in their ability to bind prenylated GTPases (Berg et al., 2010). A long form of SmgGDS that has 607 amino acids was identified and named SmgGDS-607, and the shorter form of SmgGDS that has 558 amino acids was named SmgGDS-558 (Berg et al., 2010). The functions of these splice variants were defined by using two publicly available cDNA constructs encoding SmgGDS (Berg et al., 2010). One construct encoded SmgGDS-558 that was identified in the original studies of SmgGDS (Kaibuchi et al., 1991), and the other construct encoded SmgGDS containing 607 amino acids obtained from the National Institute of Technology and Evaluation (Chiba, Japan) (Berg et al., 2010). This cDNA construct encoding SmgGDS-607 had been used in previous studies, but it was not recognized that it encoded a form of SmgGDS differing from SmgGDS-558 (Shin et al., 2006). The two SmgGDS splice variants were found to have very different abilities to bind prenylated GTPases; SmgGDS-607 binds pre-prenylated GTPases before they enter the prenylation pathway, whereas SmgGDS-558 binds only prenylated small GTPases (Berg et al., 2010; Ntantie et al., 2013; Williams, 2013; Schuld N. J. et al., 2014). This discovery that two forms of SmgGDS interact differently with pre-prenylated vs. prenylated small GTPases resolved the earlier controversy that prenylation is required for a small GTPase to bind SmgGDS.

**FIGURE 1:**
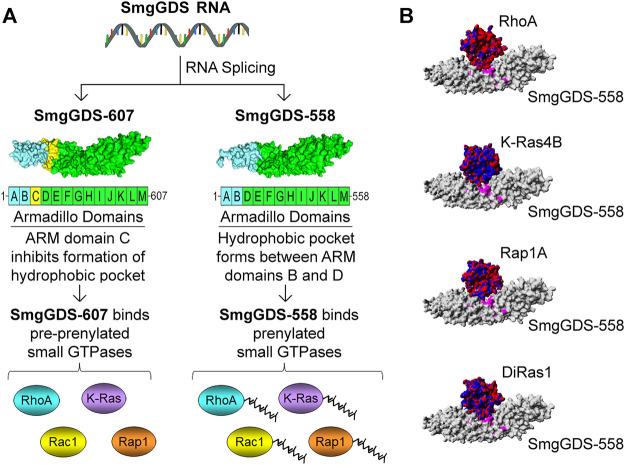
Schematic illustration depicting how the SmgGDS splice variants, SmgGDS-607 and SmgGDS-558, interact with pre-prenylated and prenylated small GTPases, respectively. **(A)** SmgGDS-607 has 13 ARM domains named A–M. SmgGDS-607 binds pre-prenylated small GTPases because the presence of ARM domain C inhibits the formation of a hydrophobic pocket in SmgGDS-607. In contrast, SmgGDS-558 lacks ARM domain C, causing it to have only 12 ARM domains. SmgGDS-558 binds prenylated small GTPases because a hydrophobic pocket forms between ARM domains B and D, which accommodates the prenyl group of small GTPases. **(B)** Homology models indicate that SmgGDS-558 binds different Ras and Rho family members in a similar manner, suggesting that these small GTPases compete for binding to SmgGDS-558. SmgGDS-558 is depicted with a gray surface plot. The small GTPases are depicted with an electrostatic surface plot with negative charges indicated by red and positive charges indicated by blue [homology models adopted from Bergom et al. (2016)].

The structural features that cause SmgGDS-607 to bind pre-prenylated GTPases and SmgGDS-558 to bind prenylated GTPases are beginning to be understood. SmgGDS is composed of multiple armadillo (ARM) domains. An ARM domain consists of approximately 40 amino acids folded into alpha helices. ARM domains can be identified from the amino acid sequence of a protein using a paradigm provided by Andrade and colleagues (Andrade et al., 2001). Using this paradigm to identify ARM domains, it was determined that SmgGDS has 13 ARM domains, which were named A–M (Berg et al., 2010). In contrast, SmgGDS-558 was reported to have only 12 ARM domains due to the absence of ARM domain C (Figure 1A) (Berg et al., 2010). The designation of these ARM domains as A–M has become the established method to describe the arrangement of ARM domains in SmgGDS (Schuld, et al., 2014a; Schuld N. J. et al., 2014; Hauser et al., 2014; Gonyo et al., 2017; Shimizu et al., 2017; Shimizu et al., 2018). In 2018, the Shimizu group solved the crystal structure of SmgGDS-558 associated with prenylated RhoA (Shimizu et al., 2018). Analysis of this structure indicates that a hydrophobic pocket that can accommodate the prenyl group of RhoA forms between ARMs B and D in SmgGDS-558. In contrast, SmgGDS-607 cannot bind prenylated GTPases because the presence of ARM C precludes the formation of this hydrophobic pocket (Shimizu et al., 2018). (Figure 1A).

Another major controversy regarding SmgGDS arose from the proposal that SmgGDS is a GEF for many different Ras and Rho family members. Early studies suggested that SmgGDS might act as a GEF for multiple PBR-containing small GTPases, including Rap1A and Rap1B (Yamamoto et al., 1990; Kaibuchi et al., 1991; Mizuno et al., 1991; Hiroyoshi et al., 1991), K-Ras4B (Mizuno et al., 1991; Orita et al., 1993; Nakanishi et al., 1994; Yaku et al., 1994), RhoA (Mizuno et al., 1991; Kikuchi et al., 1992; Yaku et al., 1994; Hutchinson et al., 2000; Hutchinson and Eccleston, 2000), Rac1 (Ando et al., 1992; Chuang et al., 1994), Rac2 (Fujioka et al., 1992; Xu et al., 1997), and Cdc42 (Yaku et al., 1994). These small GTPases bind to SmgGDS in a similar manner (Figure 1B) involving two main interactions. The PBR of the small GTPase has electrostatic interactions with an electronegative patch in SmgGDS, and the main body of the GTPase interacts with a binding groove in SmgGDS (Hamel et al., 2011). It was difficult to understand how SmgGDS could act as a GEF for so many Ras and Rho family members, because SmgGDS lacks the domains that are commonly associated with proteins that have GEF activity, including the CDC25 domain that activates Ras family members, and the DH domain that activates Rho family members. Several confounding issues hampered these earlier studies of the GEF activity of SmgGDS, including the use of crude protein preparations and long incubation times during the analysis of GDP/GTP exchange, and the fact that sophisticated methods of analyzing GEF activity were not yet widely available.

The Sondek group finally clarified the GEF activity of SmgGDS in 2011 (Hamel et al., 2011). Using real-time MANT-GDP exchange assays, these researchers demonstrated that both SmgGDS-558 and SmgGDS-607 are true GEFs for RhoA and RhoC, but they are unable to promote GDP/GTP exchange by K-Ras4B, Rap1A, Rap1B, RhoB, Rac1, Rac2, and Cdc42 (Hamel et al., 2011). Crystallographic analysis in 2018 indicated that SmgGDS promotes GDP/GTP exchange by RhoA through a unique mechanism that is not utilized by other GEFs (Shimizu et al., 2018). This analysis indicates that the switch I and switch II regions of RhoA undergo a conformational change when RhoA binds SmgGDS, which opens up the nucleotide-binding site in RhoA (Shimizu et al., 2018). This mechanism allows SmgGDS-607 and SmgGDS-558 to act as GEFs for pre-prenylated and prenylated RhoA, respectively. The incorrect statement that SmgGDS is a GEF for many small GTPases in the Ras and Rho families continues to appear in the literature and in online sources. This misleading statement should be amended to reflect our current knowledge that SmgGDS is a GEF for RhoA and RhoC, but not for other small GTPases (Hamel et al., 2011; Shimizu et al., 2018).

Even though SmgGDS has limited intrinsic GEF activity, SmgGDS still might promote the activity of many different small GTPases by serving as a scaffold that facilitates the interactions of GEFs with small GTPases bound to SmgGDS (Berg et al., 2010). The formation of a transient trimeric complex consisting of SmgGDS, a small GTPase, and the specific GEF that activates the small GTPase provides a specific mechanism for SmgGDS to increase the activities of different Ras and Rho family members. In support of this mechanism, it was reported that SmgGDS (now known to be SmgGDS-607) forms a complex with Rac1 and βPIX, which is a GEF for Rac1 (Shin et al., 2006). The association of a GEF with SmgGDS-607 provides a way to activate small GTPases before they are prenylated, since SmgGDS-607 only binds GTPases before they enter the prenylation pathway. In contrast, the association of a GEF with SmgGDS-558 will activate prenylated GTPases, since SmgGDS-558 binds small GTPases only after they have been prenylated.

## Complementary Roles of SmgGDS-607 and SmgGDS-558 in the Prenylation and Trafficking of Ras and Rho Family Members

**FIGURE 2:**
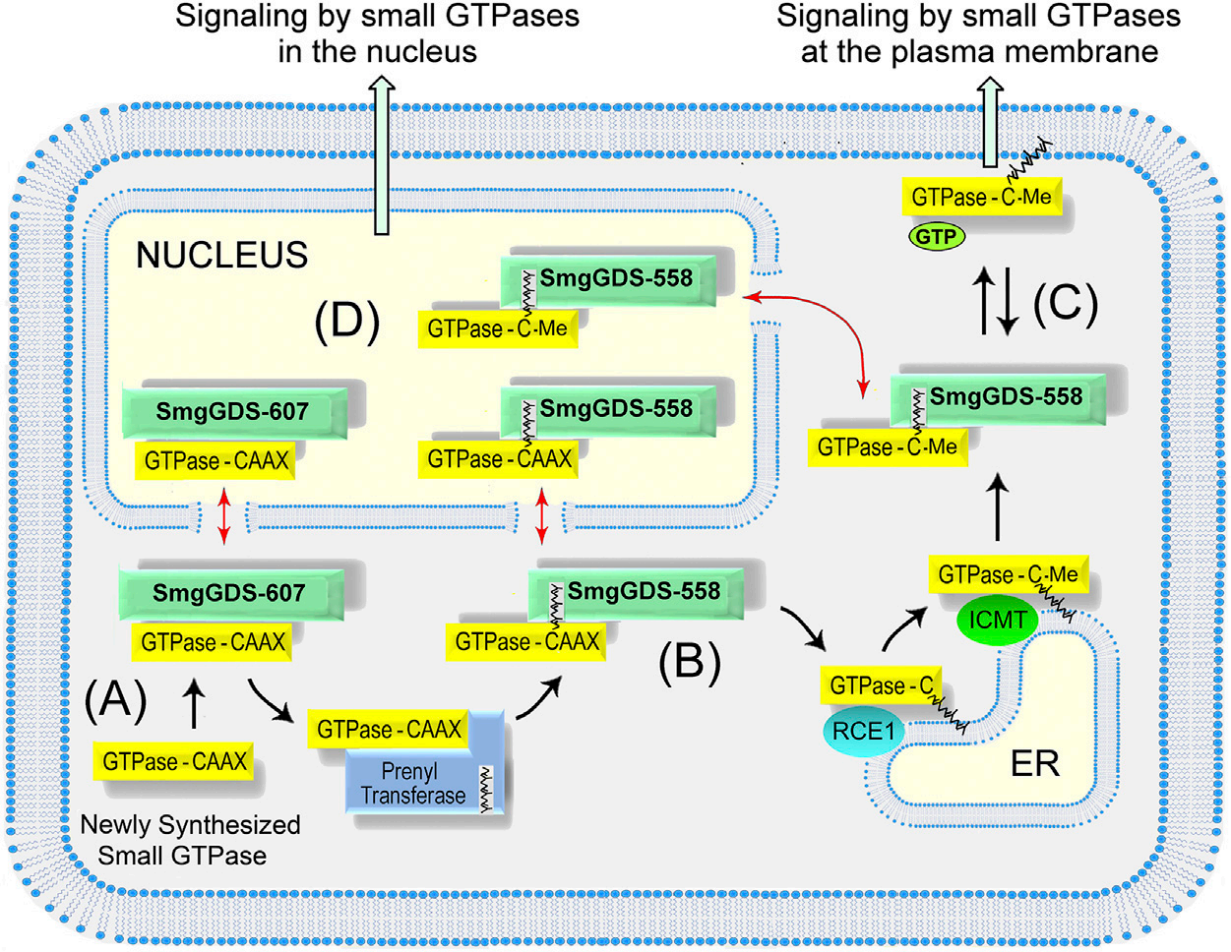
Model depicting how SmgGDS splice variants regulate the prenylation and trafficking of small GTPases. **(A)** SmgGDS-607 binds a newly synthesized small GTPase and retains it until the correct signal causes SmgGDS-607 to release the pre-prenylated GTPase to the PTase. **(B)** SmgGDS-558 escorts newly prenylated small GTPases to the ER for post-prenylation processing. **(C)** SmgGDS-558 escorts prenylated and fully processed small GTPases from the ER to the plasma membrane. **(D)** Both SmgGDS splice variants might assist in nucleocytoplasmic shuttling of small GTPases (red arrows).

Multiple studies indicate that SmgGDS-607 and SmgGDS-558 work together to regulate the prenylation and trafficking of small GTPases in the Ras and Rho families (Figure 2) (Berg et al., 2010; Williams 2013; Schuld N. J. et al., 2014; Brandt et al., 2020). SmgGDS-607 binds newly synthesized small GTPases that possess a PBR and regulates their entry into the prenylation pathway (Figure 2A). It was originally proposed that SmgGDS-607 acts as a gatekeeper for small GTPases entering the prenylation pathway (Berg et al., 2010). Just as a gatekeeper has the power to open a gate but also to lock it shut, it was suggested that SmgGDS-607 can help small GTPases gain access to the prenylation pathway but also restrain small GTPases from inappropriately entering the prenylation pathway (Berg et al., 2010). This proposed role of SmgGDS-607 as a gatekeeper for the prenylation pathway is supported by reports that SmgGDS-607 can both facilitate (Berg et al., 2010; Ntantie et al., 2013; Garcia-Torres and Fierke, 2019; Nissim et al., 2019; Brandt et al., 2020) and suppress (Berg et al., 2010; Jennings et al., 2018; Garcia-Torres and Fierke, 2019) the prenylation of small GTPases that bind SmgGDS-607.

SmgGDS-607 recognizes the last amino acid in the CAAX motif of the GTPase, preferring to interact with small GTPases that have a CAAX motif ending in leucine rather than methionine (Schuld N. J. et al., 2014). This finding suggests that SmgGDS-607 preferentially binds small GTPases that are destined to become geranylgeranylated by GGTase-I, since GGTase-I prenylates small GTPases with a CAAX motif ending in leucine. Despite this preference for GTPases that will be geranylgeranylated, SmgGDS-607 also binds small GTPases that have a CAAX motif ending in methionine (Schuld N. J. et al., 2014; Garcia-Torres and Fierke, 2019; Nissim et al., 2019), which will be farnesylated by FTase, indicating that SmgGDS-607 probably regulates the prenylation of both geranylgeranylated and farnesylated small GTPases. The ability of SmgGDS-607 to deliver pre-prenylated Ras and Rho family members to PTases indicates that SmgGDS-607 has functional similarities to REP1, which delivers pre-prenylated Rab family members to RabGGTase (Preising and Ayuso, 2004; Goody et al., 2005; Wu et al., 2007).

SmgGDS-558 differs significantly from SmgGDS-607 because SmgGDS-558 binds only prenylated small GTPases (Berg et al., 2010; Williams 2013; Schuld N. J. et al., 2014). SmgGDS-558 may intercept PBR-containing small GTPases after they have been prenylated by the PTase and help them traffic to the ER for post-prenylation processing (Figure 2B). For this interaction to occur, SmgGDS-558 must bind prenylated small GTPases before the C-terminal AAX sequence is cleaved during post-prenylation processing. The ability of SmgGDS-558 to bind prenylated GTPases that retain the AAX sequence is supported by the finding that SmgGDS-558 binds small GTPases that were produced in reticulocyte lysates containing PTases but lacking the membrane-associated enzyme needed for post-prenylation processing (Lanning et al., 2004; Bergom et al., 2016). Additionally, the Shimizu group solved the crystal structure of SmgGDS-558 bound to prenylated RhoA that still retained the AAX sequence because it had not undergone post-prenylation processing (Shimizu et al., 2018). SmgGDS-558 may help newly prenylated GTPases arrive at the ER membrane or facilitate their interactions with RCE1 and ICMT, which remove the AAX sequence and carboxylmethylate the prenylated GTPase at the ER membrane (Figure 2B). These proposed interactions of SmgGDS-558 with newly prenylated GTPases in the Ras and Rho families are functionally similar to the interactions of REP1 with newly prenylated GTPases in the Rab family (Preising and Ayuso, 2004; Goody et al., 2005; Leung et al., 2006; Wu et al., 2007).

It is likely that SmgGDS-558 also acts as a chaperone that helps prenylated small GTPases move to the plasma membrane or other regions of the cell after post-prenylation processing has been completed at the ER (Figure 2C) SmgGDS-558 has a hydrophobic pocket that can shield the prenyl group of small GTPases (Shimizu et al., 2018) moving through the aqueous cytosol. Chaperones that shield the prenyl groups of different Ras and Rho family members include PDEδ (Bhagatji et al., 2010; Dharmaiah et al., 2016), PRA1 (Figueroa et al., 2001; Bhagatji et al., 2010), VPS35 (Zhou et al., 2016), and RhoGDI (Garcia-Mata et al., 2011). The chaperone for prenylated Rab proteins is RabGDI (Preising and Ayuso, 2004; Goody et al., 2005; Leung et al., 2006; Wu et al., 2007). Each of these chaperones may have specialized functions. For example, PDEδ helps farnesylated Ras family members such as K-Ras4B move between the plasma membrane and endomembranes (Bhagatji et al., 2010; Dharmaiah et al., 2016), whereas RhoGDI helps geranylgeranylated Rho family members such as RhoA and Rac1 move mainly between the plasma membrane and the cytoplasm (Garcia-Mata et al., 2011). SmgGDS-558 may share multiple functions with these chaperones, since SmgGDS-558 binds multiple Ras and Rho family members that are farnesylated or geranylgeranylated.

In addition to escorting prenylated small GTPases to the plasma membrane, it is likely that SmgGDS-558 also escorts prenylated GTPases into the nucleus (Figure 2D). Nucleocytoplasmic shuttling by SmgGDS-558 was discovered in 2003, when it was found to have a N-terminal nuclear export sequence and to accumulate with Rac1 in the nucleus (Lanning et al., 2003). The PBR of Rac1 was discovered to function as a nuclear localization sequence, and exchanging the PBR of Rac1 with the PBR of RhoA, which lacks an NLS, inhibits the nuclear accumulation of Rac1 (Lanning et al., 2003; Lanning et al., 2004). Subsequent studies confirmed the nuclear accumulation of prenylated Rac1 (Michaelson et al., 2008). Several functions of nuclear Rac1 have been described, including controlling nuclear shape (Navarro-Lerida et al., 2015), stimulating rRNA synthesis (Justilien et al., 2017), promoting the cell cycle (Michaelson et al., 2008), inducing neoplastic transformation (Huff et al., 2013), and enhancing malignancy (Huff et al., 2013; Navarro-Lerida et al., 2015; Justilien et al., 2017). Rac1 is activated in the nucleus by the GEF ECT2 (Huff et al., 2013; Justilien et al., 2017), and it is inactivated by a nuclear variant of β1-chimaerin (Casado-Medrano et al., 2020). The binding of prenylated Rac1 to SmgGDS-558 provides a way for prenylated Rac1 to enter the nucleus and participate in these signaling pathways.

While SmgGDS-558 serves as a nuclear chaperone for prenylated small GTPases, SmgGDS-607 might serve as a nuclear chaperone for pre-prenylated GTPases (Figure 2D). Pre-prenylated small GTPases can exhibit significant nuclear accumulation (Roberts et al., 2008; Lee et al., 2012; Ntantie et al., 2013; Navarro-Lerida et al., 2015; Wilson et al., 2015). Many small GTPases accumulate in the nucleus when they are maintained in the pre-prenylated state due to pharmacological inhibition of PTases or mutation of the cysteine in the CAAX motif (Lee et al., 2012; Navarro-Lerida et al., 2015; Wilson et al., 2015). The lack of a prenyl group will keep GTPases from anchoring at membranes, which might cause pre-prenylated GTPases to diffuse passively into the nucleus due to their small size (∼21 kDa). The binding of a small GTPase to SmgGDS-607 provides a specific mechanism to control the nuclear entry of small GTPases before they are prenylated. SmgGDS-607 might serve as a chaperone that keeps pre-prenylated GTPases from inappropriately entering the nucleus, or alternatively SmgGDS-607 may actively promote the nuclear entry of some GTPases before they are prenylated. In addition to controlling entry into the nucleus, SmgGDS-558 and SmgGDS-607 might also utilize their N-terminal nuclear export sequence (Lanning et al., 2003) to escort small GTPases out of the nucleus and return them to the cytoplasm when nuclear signaling is completed (Figure 2D).

## Signaling Events and Protein Partners of SmgGDS Control the Prenylation and Trafficking of Ras and Rho Family Members

Events that alter the interactions of SmgGDS with Ras and Rho family members are being recognized as important regulatory mechanisms that control the prenylation and trafficking of these small GTPases. When SmgGDS-607 binds a newly synthesized small GTPase, SmgGDS-607 may retain the small GTPase until the correct signal releases the small GTPase into the prenylation pathway (Berg et al., 2010; Schuld N. J. et al., 2014; Jennings et al., 2018; Garcia-Torres and Fierke, 2019). The signals that release a GTPase from SmgGDS-607 will determine when the GTPase will be prenylated, since the CAAX motif of the GTPase is inaccessible to the PTase as long as the GTPase is bound to SmgGDS-607 (Schuld N. J. et al., 2014). The major signal that releases a GTPase from SmgGDS-607 is thought to be GDP/GTP exchange (Berg et al., 2010), which could be stimulated by a GEF that interacts with the GTPase bound to SmgGDS-607 or by SmgGDS-607 acting as a direct GEF for RhoA or RhoC (Berg et al., 2010; Hamel et al., 2011; Jennings et al., 2018). The report that SmgGDS (now known to be SmgGD-607) forms a complex with Rac1 and the GEF βPIX (Shin et al., 2006) indicates that SmgGDS-607 can facilitate GDP/GTP exchange by bringing small GTPases into contact with their specific GEFs. It was found that GDP/GTP exchange accelerates the prenylation of Rap1 in cells (Berg et al., 2010) but the identities of the GEFs that initiate the prenylation of Rap1 or other GTPases have not yet been determined.

**FIGURE 3:**
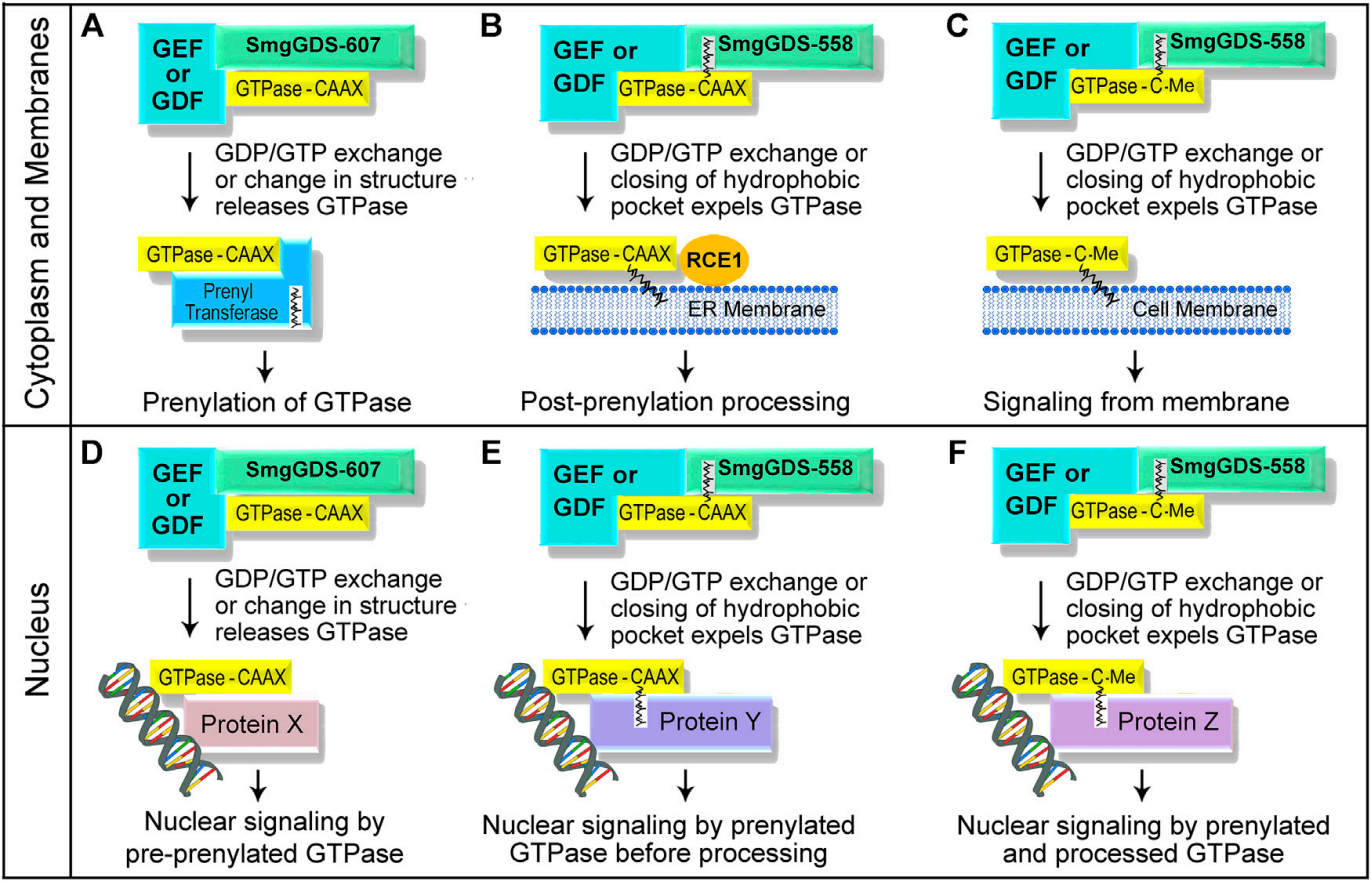
Schematic illustration depicting how unidentified GEFs and GDFs might release small GTPases from SmgGDS splice variants in the cytoplasm and at membranes **(A–C)**, and in the nucleus **(D–F)**. The interactions of these proteins with SmgGDS will control when the small GTPases will be prenylated or undergo post-prenylation processing, and determine where the small GTPases will localize in the cell.

There are over 100 GEFs located in the cytoplasm, nucleus, and at the plasma membrane that can activate members of the Ras and Rho families (Vigil et al., 2010; Gray et al., 2020), and a small GTPase that is bound to SmgGDS-607 may interact with its GEFs in these different regions of the cell. Since SmgGDS is a nucleocytoplasmic shuttling protein that associates with small GTPases in both the cytoplasm and the nucleus (Lanning et al., 2003; Lanning et al., 2004; Gonyo et al., 2017), a pre-prenylated small GTPase that is bound to SmgGDS-607 is likely to encounter both cytoplasmic and nuclear GEFs. If a pre-prenylated GTPase that is bound to SmgGDS-607 encounters its GEF in the cytoplasm, the released GTPase can interact with the cytoplasmic PTase and become prenylated (Figure 3A). In contrast, if a pre-prenylated GTPase that is bound to SmgGDS-607 encounters its GEF in the nucleus, the GTPase may be released from SmgGDS-607 in the nucleus, where it may remain in a pre-prenylated state due to the absence of PTases in the nucleus (Figure 3D). More studies are needed to define how prenylation is controlled by GEFs that interact with GTPases bound to SmgGDS-607 in different regions of the cell.

Similar to the mechanisms that regulate SmgGDS-607, specific signaling events may control the ability of SmgGDS-558 to deliver and release prenylated small GTPases at specific sites in the cell. Certain signals may direct SmgGDS-558 to the ER membrane, the plasma membrane, or to the nucleus when a prenylated GTPase is bound to SmgGDS-558. The prenylated GTPase may be released from SmgGDS-558 at these sites when it encounters its GEF and undergoes GDP/GTP. By releasing a GTPase from SmgGDS-558, these GEFs will control when the small GTPase will undergo post-prenylation processing (Figure 3B) and where it will localize in the cell (Figures 3C,E,F). The specific GEFs that release prenylated GTPases from SmgGDS-558 have not yet been identified, but likely candidates include ECT2, Net1, and RapGEF5 which are GEFs that promote GDP/GTP exchange by different Ras and Rho family members in the nucleus and the cytoplasm (Dubash et al., 2011; Huff et al., 2013; Justilien et al., 2017; Griffin et al., 2018). Prenylated GTPases might be released from SmgGDS-558 at the plasma membrane when they encounter membrane-localized GEFs (Figure 3C), which will promote membrane association of the GTPases and their participation in signaling cascades at the plasma membrane.

In addition to GEFs, proteins called GDI displacement factors (GDFs) might also release prenylated GTPases from SmgGDS-558 (Figure 3). There are several known GDFs that release prenylated GTPases from chaperones such as RabGDI (Dirac-Svejstrup et al., 1997; Collins, 2003; Sivars et al., 2003; Ismail, 2017) and PDEδ (Ismail et al., 2011; Williams, 2011; Dharmaiah et al., 2016; Fansa and Wittinghofer, 2016; Ismail, 2017; Kuchler et al., 2018). Two well characterized GDFs that release farnesylated Ras family members from PDEδ are Arl2 and Arl3, which are members of the Arf family of small GTPases. Arl2 or Arl3 binds PDEδ when a farnesylated Ras family member is also bound to PDEδ, forming a trimeric complex. When the GTP-bound form of Arl2 or Arl3 binds PDEδ, the hydrophobic pocket of PDEδ becomes so narrow that the farnesylated Ras family member is expelled from PDEδ (Ismail et al., 2011; Williams 2011). The farnesylated GTPase that is expelled from PDEδ associates with membranes, where it participates in membrane-localized signaling cascades (Ismail et al., 2011; Williams, 2011; Fansa and Wittinghofer, 2016; Ismail, 2017; Kuchler et al., 2018). It is probable that specific GDFs induce SmgGDS-558 to release prenylated GTPases at membranes (Figures 3B,C) or in the nucleus (Figures 3E,F). GDF-like proteins may also induce SmgGDS-607 to release pre-prenylated GTPases to PTases (Figure 3A) or to nuclear proteins (Figure 3D).

Recent studies have identified two abnormal Rab proteins that might serve as GDFs for SmgGDS. These proteins consist of the N-terminal portions of RabL3 (Nissim et al., 2019) or Rab22a (Liao et al., 2020), and exhibit enhanced binding to SmgGDS-607 and SmgGDS-558 in pancreatic cancer (Nissim et al., 2019) and osteosarcoma (Liao et al., 2020), respectively, and are also detected in breast cancer (Nissim et al., 2019; Liao et al., 2020). These abnormal Rab proteins bind to SmgGDS when a member of the Ras or Rho family is also bound to SmgGDS, forming a trimeric complex (Nissim et al., 2019; Liao et al., 2020). The abnormal RabL3 protein that occurs in familial pancreatic cancer is a truncated protein consisting of the first 1–36 amino acids of RabL3, designated RabL3^1–36^ (Nissim et al., 2019). This truncated RabL3^1–36^ protein binds to SmgGDS-607 when K-Ras4B is bound, which increases the prenylation and membrane trafficking of K-Ras4B (Nissim et al., 2019). These findings suggest that RabL3^1–36^ acts as a GDF that binds SmgGDS-607 when pre-prenylated K-Ras4B is also bound, promoting the release of K-Ras4B to the prenyltransferase and accelerating K-Ras4B prenylation, similar to the mechanism depicted in Figure 3A. The RabL3^1–36^ protein might also serve as a GDF for SmgGDS-558, similar to the mechanism depicted in Figure 3C, because RabL3^1–36^ forms a trimeric complex with SmgGDS-558 and K-Ras4B and accelerates the accumulation of newly synthesized K-Ras4B at membranes (Nissim et al., 2019).

In contrast to the RabL3^1–36^ protein that arises by truncation (Nissim et al., 2019), the abnormal Rab22a proteins that occur in osteosarcoma are fusion proteins consisting of the first 1–38 amino acids of Rab22a followed by various sequences encoded by different regions of chromosome 20 (Liao et al., 2020). The Rab22a^1–38^ portion of these fusion proteins binds to SmgGDS-607 when RhoA is bound (Liao et al., 2020). The formation of this trimeric complex accelerates the release of RhoA from SmgGDS-607, increases GTP-binding by RhoA, and enhances membrane localization of RhoA (Liao et al., 2020). Since only the pre-prenylated form of RhoA binds to SmgGDS-607 (Berg et al., 2010), it is likely that Rab22a^1–38^ promotes the prenylation of RhoA by releasing pre-prenylated RhoA from SmgGDS-607 to the prenyltransferase (Figure 3A). However, the effect of Rab22a^1–38^ on the prenylation of RhoA has not yet been determined. Intriguingly, both RabL3^1–36^ and Rab22a^1–38^ interact with several Ras and Rho family members in addition to K-Ras4B and RhoA (Nissim et al., 2019; Liao et al., 2020). It was also reported that RabL3^1–36^ and Rab22a^1–38^ interact with both SmgGDS-607 and SmgGDS-558 (Nissim et al., 2019; Liao et al., 2020). These features suggest that RabL3^1–36^ and Rab22a^1–38^ may have broad roles as GDFs for multiple Ras and Rho family members that associate with SmgGDS-607 and SmgGDS-558.

In contrast to these mutant Rab proteins, which promote cancer by forming trimeric complexes with SmgGDS and an oncogenic small GTPase (Nissim et al., 2019; Liao et al., 2020), the GTPase DiRas1 (also known as Rig) seems to inhibit cancer by blocking the binding of small GTPases to SmgGDS. DiRas1 is a Ras family member that acts as a tumor suppressor in many types of cancer (reviewed in Li et al., 2019). DiRas1 binds to SmgGDS (Bergom et al., 2016; Gonyo et al., 2017; Garcia-Torres and Fierke, 2019) (Figure 1B) and inhibits the binding of other small GTPases, including RhoA, K-Ras4B, and Rap1A (Bergom et al., 2016). *In silico* docking indicates that DiRas1 directly competes with other small GTPases for binding to SmgGDS (Bergom et al., 2016), and DiRas1 binds with much stronger affinity than other Ras and Rho family members to SmgGDS-558 (Bergom et al., 2016) and to SmgGDS-607 (Garcia-Torres and Fierke, 2019). In cancer cells, ectopic expression of DiRas1 inhibits basal and RhoA-mediated NF-kB activity (Bergom et al., 2016) and provokes responses that can be attributed to reduced signaling by Ras and Rho family members [reviewed in Li et al. (2019)]. Ectopic expression of DiRas1 also alters nucleocytoplasmic shuttling of SmgGDS-558 and diminishes its interaction with UBF in the nucleus (Gonyo et al., 2017). These findings support the model that DiRas1 acts as a tumor suppressor by inhibiting the binding of other small GTPases to SmgGDS-607 and SmgGDS-558. DiRas1 is expressed in normal cells, and the binding of DiRas1 to SmgGDS in these cells may suppress SmgGDS interactions with Ras and Rho family members and keep the activity of these GTPases in check. In contrast, the loss of DiRas1 expression in malignant cells removes this brake, allowing SmgGDS to interact with Ras and Rho family members and promote their oncogenic activities (Bergom et al., 2016).

Taken together, these findings indicate that different GEFs, GDFs, and other proteins such as DiRas1 may regulate the interactions of SmgGDS-607 and SmgGDS-558 with pre-prenylated and prenylated GTPases, respectively, in different regions of the cell (Figure 3). These interactions will have profound effects on the prenylation, trafficking, and signaling by Ras and Rho family members (Figure 3). Future studies are needed to characterize the functions of the abnormal Rab proteins that might act as GDFs for SmgGDS (Nissim et al., 2019; Liao et al., 2020), and to characterize GEFs and other proteins that control the interactions of small GTPases with SmgGDS.

Post-translational modification of either SmgGDS or its small GTPase partner is another event that may alter the interactions between these proteins and affect the prenylation and trafficking of the small GTPase. Post-translational modifications of SmgGDS have not been well characterized. However, signaling cascades that promote the phosphorylation of serines in the PBR of small GTPases have been found to alter the prenylation of small GTPases (Ntantie et al., 2013; Williams, 2013; Wilson et al., 2015; Wilson et al., 2016). The binding of small GTPases to SmgGDS-607 depends on the electrostatic charge of the PBR (Hamel et al., 2011), and diminishing this charge by phosphorylation may diminish interactions with SmgGDS-607. The small GTPases K-Ras4B, Rap1A, Rap1B, and RhoA have serines in their PBRs that can be phosphorylated (reviewed in Williams, 2013), but Rap1B is the GTPase that seems to be most sensitive to phosphorylation-dependent regulation of prenylation (Ntantie et al., 2013; Williams, 2013; Wilson et al., 2015; Wilson et al., 2016).

Activation of A2B adenosine receptors or *β*-adrenergic receptors causes protein kinase A to phosphorylate two serines in the PBR of Rap1B before it is prenylated (Ntantie et al., 2013; Wilson et al., 2015; Wilson et al., 2016). This phosphorylation diminishes interactions of newly synthesized Rap1B with SmgGDS-607, suppressing Rap1B prenylation and causing pre-prenylated Rap1B to accumulate in the cytoplasm and nucleus (Ntantie et al., 2013; Wilson et al., 2015). The absence of prenylated Rap1B at the plasma membrane diminishes Rap1B-mediated cell–cell adhesion (Ntantie et al., 2013; Wilson et al., 2015), and the nuclear accumulation of pre-prenylated Rap1B may promote events that are known to be regulated by nuclear Rap1B, including signaling by *β*-catenin (Goto et al., 2010; Griffin et al., 2018). Together, these events induce cell scattering and promote the metastatic phenotype (Ntantie et al., 2013; Wilson et al., 2015). The finding that Rap1B prenylation is reduced in rat mammary tumors provides additional evidence that this pathway has a role in cancer (Ntantie et al., 2013). These findings indicate that chronic exposure of cancer cells to adenosine and norepinephrine in the tumor microenvironment may enhance metastasis by chronically suppressing Rap1B prenylation (Ntantie et al., 2013; Williams, 2013; Wilson et al., 2015; Wilson et al., 2016). There are undoubtedly many more undiscovered signaling cascades that control prenylation by regulating the interactions of small GTPases with SmgGDS-607.

## Therapeutic Targeting of SmgGDS in Cancer

SmgGDS has a well-established role in cancer progression. SmgGDS expression is increased in breast, lung, and prostate cancer (Tew et al., 2008; Zhi et al., 2009; Hauser et al., 2014), and elevated SmgGDS expression is associated with poor prognosis in breast cancer (Hauser et al., 2014). SmgGDS promotes cell proliferation, migration, and NF-kB activity in breast, lung, prostate, and pancreatic cancer lines (Tew et al., 2008; Zhi et al., 2009; Berg et al., 2010; Schuld N. J. et al., 2014; Hauser et al., 2014; Gonyo et al., 2017; Brandt et al., 2020) and promotes tumorigenesis of human breast cancer and lung cancer xenografts in mouse models (Schuld N. et al., 2014; Hauser et al., 2014). Early studies of SmgGDS in cancer did not differentiate between SmgGDS-607 and SmgGDS-558 (Tew et al., 2008; Zhi et al., 2009), making it difficult to discern the roles of each splice variant. However, more recent studies have determined that the generation of SmgGDS-607 and SmgGDS-558 is uniquely regulated in cancer cells (Brandt et al., 2020), and both splice variants contribute to malignancy (Berg et al., 2010; Schuld N. et al., 2014; Hauser et al., 2014; Gonyo et al., 2017; Brandt et al., 2020).

An oncogenic splicing program that generates much more SmgGDS-607 than SmgGDS-558 occurs in breast and lung cancer (Brandt et al., 2020). A high ratio of SmgGDS-607: SmgGDS-558 (referred to as the 607:558 ratio) occurs in cells that are rapidly proliferating and migrating, and tissues that contain more proliferative and migratory cells have a higher 607:558 ratio (Brandt et al., 2020). For example, the 607:558 ratio is approximately 2:1 in the mouse spleen, which has a high proportion of cells that proliferate and migrate. In contrast, the 607:558 ratio is approximately 1:3 in the mouse brain, which contains mainly terminally differentiated, non-migratory cells. Most notably, the 607:558 ratio is highest in cancer cell lines, reaching a value of approximately 8:1 (Brandt et al., 2020). Additional evidence that a high 607:558 ratio is associated with malignancy is provided by the finding that the 607:558 ratio increases as mammary tumors develop in rat and mouse models, and a high 607:558 ratio in patients’ breast tumors is associated with reduced survival (Brandt et al., 2020).

The very high 607:558 ratio in cancer cells may be related to the increased expression and diversity of Ras and Rho family members needed to maintain the malignant phenotype. The rapid proliferation and migration of cancer cells depends on signaling cascades regulated by many different Ras and Rho family members, resulting in increased expression of small GTPases in the Ras and Rho families in malignant cells (Gómez del Pulgar et al., 2005; Konstantinopoulos et al., 2007; Alan and Lundquist, 2013; Haga and Ridley, 2016; Porter et al., 2016; Wong et al., 2018). Cancer cells may require an elevated amount of SmgGDS-607 to bind the excessive number of newly synthesized small GTPases and facilitate their entry into the prenylation pathway. There is less of a need for SmgGDS-558 than for SmgGDS-607, because SmgGDS-558 intercepts only the proportion of small GTPases that have been released by SmgGDS-607 and have become prenylated. Despite requiring less SmgGDS-558 than SmgGDS-607, cancer cells still need a threshold level of SmgGDS-558, as indicated by reports that the RNAi-mediated depletion of SmgGDS-558 significantly diminishes malignancy (Berg et al., 2010; Schuld N. et al., 2014; Hauser et al., 2014).

**FIGURE 4:**
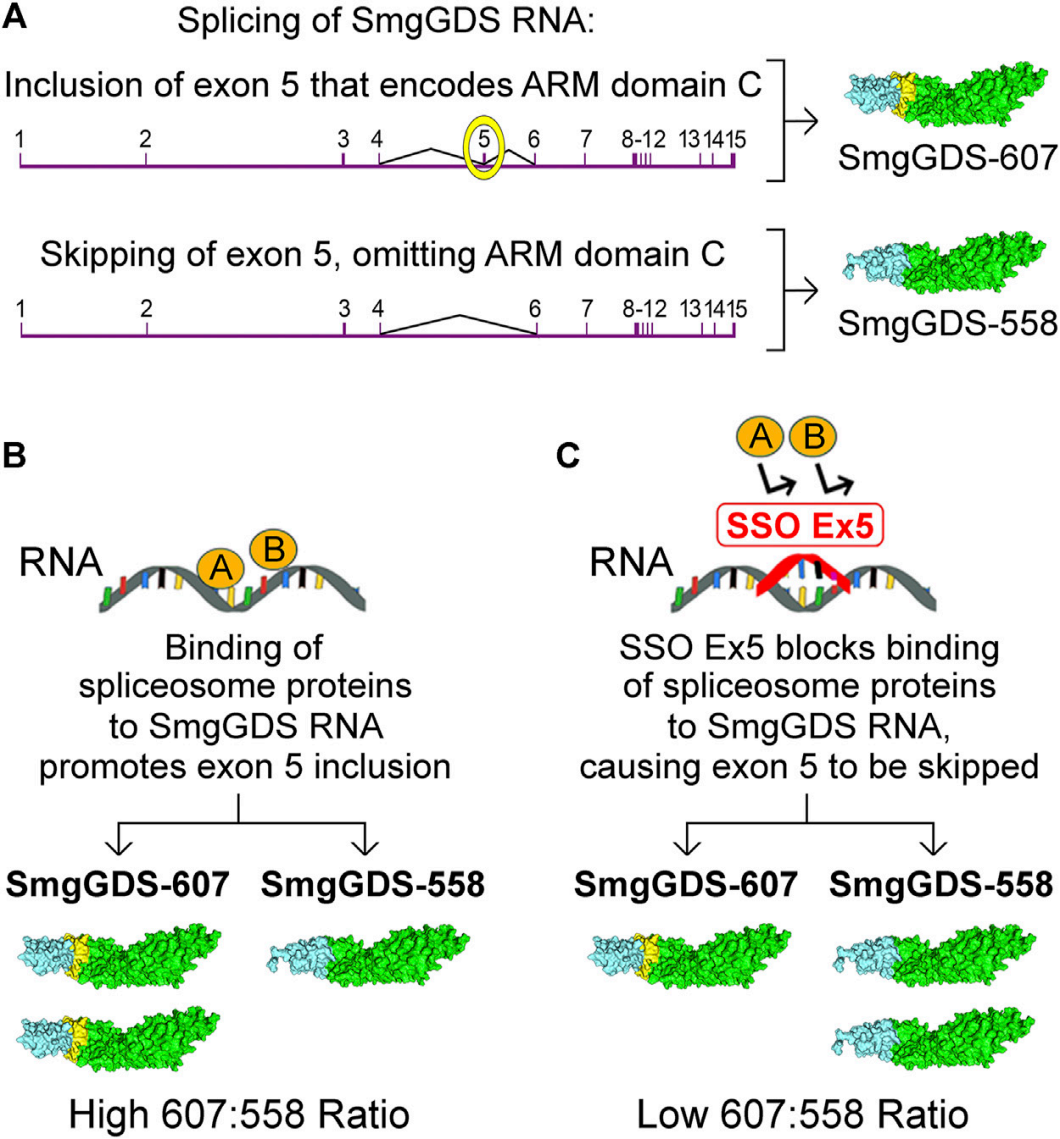
Schematic illustration depicting the regulation of SmgGDS expression by the splice-switching oligonucleotide, SSO Ex5. **(A)** SmgGDS RNA contains 15 exons, and exon 5 encodes ARM domain C. Inclusion of exon 5 in mature SmgGDS mRNA generates SmgGDS-607, whereas omission of exon 5 in mature SmgGDS mRNA generates SmgGDS-558. **(B)** In cancer cells, the binding of unspecified spliceosome proteins to SmgGDS RNA promotes exon 5 inclusion and generates more SmgGDS-607 than SmgGDS-558. **(C)** Binding of SSO Ex5 to SmgGDS RNA promotes exon 5 skipping, generating more SmgGDS-558 than SmgGDS-607. Additional manuscript sections.

The high 607:558 ratio in cancer cells offers a unique therapeutic opportunity to diminish malignancy. Splice-switching oligonucleotides (SSOs) that restore normal splicing are providing new therapies for cancer and other diseases (Havens and Hastings, 2016; El Marabti and Younis, 2018; Bonnal et al., 2020). The value of disrupting SmgGDS RNA splicing as a therapeutic option is demonstrated by the development of SSO Ex5, which is an SSO that lowers the high 607:558 ratio in cancer cells (Brandt et al., 2020). SSO Ex5 was developed by targeting the splicing events that generate SmgGDS-607 and SmgGDS-558 (Figure 4). SmgGDS-607 is generated when mature SmgGDS mRNA contains exon 5, which is the exon that encodes ARM C that is present only in SmgGDS-607 (Figure 4A). In contrast, SmgGDS-558 is generated when exon 5 is skipped during splicing of SmgGDS pre-mRNA (Figure 4A). The binding of currently undefined spliceosome proteins to SmgGDS pre-mRNA causes inclusion of exon 5, resulting in greater expression of SmgGDS-607 than SmgGDS-558 and a high 607:558 ratio (Figure 4B). When SSO Ex5 binds to SmgGDS pre-mRNA, SSO Ex5 blocks these spliceosome proteins and forces skipping of exon 5, which decreases SmgGDS-607 expression and increases SmgGDS-558 expression, lowering the 607:558 ratio (Figure 4C) (Brandt et al., 2020).

SSO Ex5 suppresses the prenylation of multiple Ras and Rho family members in cancer cells, consistent with SSO Ex5 reducing SmgGDS-607 expression (Brandt et al., 2020). This extensive loss of prenylation is accompanied by a broad range of effects, including changes in RNA expression indicating loss of signaling by Rac, RhoA, PI3K/AKT, and ERK/MAPK. Treatment of cancer cells with SSO Ex5 induces endoplasmic reticulum stress and the unfolded protein response, and ultimately causes apoptosis (Brandt et al., 2020). In addition to decreased SmgGDS-607 expression, it is likely that increased SmgGDS-558 expression also contributes to the effects of SSO Ex5. The excessive increase in the amount of SmgGDS-558 caused by SSO Ex5 might solubilize prenylated GTPases from membranes, due to cytosolic SmgGDS-558 capturing prenylated GTPases as they dissociate from membranes. Additionally, cells treated with SSO Ex5 may have more complexes of free SmgGDS-558 that can capture prenylated GTPases from membranes, because reduced prenylation will decrease the number of newly prenylated GTPases that normally bind to SmgGDS-558. Previous studies indicate that ectopic expression of SmgGDS-558 can solubilize prenylated GTPases from membranes (Kawamura et al., 1991; Kawamura et al., 1993; Nakanishi et al., 1994), and overexpression of SmgGDS-558 was found to promote apoptosis of cancer cells (Brandt et al., 2020). These results indicate that SSO Ex5 most likely inhibits malignancy by the combined effects of decreased SmgGDS-607 expression and increased SmgGDS-558 expression. The potential therapeutic value of SSOs that disrupt SmgGDS expression is indicated by the finding that intraperitoneal injection of SSO Ex5 diminishes mammary tumorigenesis in the aggressive MMTV-PyMT mouse model, without causing detectable deleterious side-effects in the mice (Brandt et al., 2020).

In addition to SSOs, other strategies to inhibit SmgGDS functions in cancer are beginning to be developed. Chemical inhibitors of SmgGDS have not been reported, but a peptide inhibitor that targets SmgGDS-607 has recently been described (Liao et al., 2020). The Kang laboratory generated a cell-penetrating synthetic peptide corresponding to the first 1–10 amino acids in Rab22a, based on their discovery that fusion proteins containing Rab22a^1–38^ bind SmgGDS-607 in osteosarcoma (Liao et al., 2020). They found that this peptide binds to SmgGDS-607, blocks interactions of SmgGDS with Rab22a^1–38^, decreases RhoA activity, and reduces cell migration and invasion. Furthermore, this peptide inhibitor diminishes lung metastases of osteosarcoma in a mouse model, and increases survival time of the mice bearing the tumors (Liao et al., 2020). These findings provide further evidence for the important role of SmgGDS in malignancy, and highlight the value of developing agents to target SmgGDS-607 and SmgGDS-558 in cancer. SmgGDS has recently been recognized to play a role in other disorders such as neurological deficits (Asiri et al., 2020), abnormal vascular branching (Wang et al., 2017), and development of aortic aneurysms (Nogi et al., 2018; Renard, 2018), indicating that the therapeutic targeting of SmgGDS should extend beyond our current efforts focused on cancer.

## Future Directions

The importance of SmgGDS throughout the animal kingdom is indicated by phylogenetic analyses suggesting that it was present in the last common eukaryotic ancestor that existed over 500 million years ago (Gul et al., 2017). The expression of SmgGDS was maintained during metazoan development, and its functions have become more diverse and complex as animals evolved. The discovery of two complementary but distinctly different splice variants of SmgGDS that regulate the prenylation and trafficking of Ras and Rho family members has defined SmgGDS as a master regulator of these small GTPases. Despite our growing understanding of how SmgGDS interacts with these small GTPases, many questions remain. Some of these questions and critical focal points for future studies are included in the following list:

• *How do cells regulate the expression and activity of SmgGDS-607 and SmgGDS-558?*
The balanced expression of SmgGDS-607 and SmgGDS-558 in cells is regulated through specific splicing programs and spliceosome factors that have yet to be characterized. Additionally, cells control the activities of these splice variants through the actions of DiRas (Bergom et al., 2016), which is expressed in normal cells [reviewed by Li et al. (2019)], and by the actions of mutant forms of both RabL3 and Rab22, which are expressed in cancer cells (Nissim et al., 2019; Liao et al., 2020). There are undoubtedly more regulatory mechanisms that control the expression, stability, and activity of these SmgGDS splice variants in different physiological and pathophysiological conditions.
• *How do post-translational modifications of the SmgGDS splice variants affect their abilities to regulate small GTPases?*
Online databases such as PhosphoSitePlus® indicate that SmgGDS has multiple residues that are ubiquitinated, acetylated, or phosphorylated. Our understanding of how SmgGDS-607 and SmgGDS-558 might be post-translationally modified and how these modifications might affect SmgGDS functions is still very rudimentary.
• *Which small GTPases interact with SmgGDS, and what are the functional consequences of these interactions?*
SmgGDS preferentially binds small GTPases that contain a PBR, including RhoA, RhoC, Rac1, Cdc42, K-Ras4A, Rap1A, Rap1B, and DiRas1, as discussed above. SmgGDS probably binds many more PBR-containing small GTPases (Table 1), and these interactions may have multiple effects. In most cases, the binding of a small GTPase to SmgGDS regulates the prenylation and trafficking of the bound GTPase (Figure 2). However, some small GTPases control the activity of SmgGDS. For example, DiRas1 inhibits SmgGDS functions (Bergom et al., 2016), whereas RabL3^1–36^ (Nissim et al., 2019), Rab22a^1–38^ (Liao et al., 2020) and potentially wildtype Rab proteins might act as GDFs that control the ability of SmgGDS to release small GTPases in different locations in the cell. More studies are needed to clarify these interactions.
• *Which signaling pathways control the prenylation and trafficking of small GTPases by altering their interactions with SmgGDS?*
Activation of A2B adenosine receptors and *β*-adrenergic receptors promotes phosphorylation of serines in the PBR of pre-prenylated Rap1B. This phosphorylation of the PBR disrupts the interactions of pre-prenylated Rap1B with SmgGDS-607, suppressing the prenylation of Rap1B and causing it to accumulate in the nucleus instead of localizing at the cell membrane (Ntantie et al., 2013; Wilson et al., 2015; Wilson et al., 2016). Other small GTPases also have serines in their PBR that can be phosphorylated [reviewed in Williams (2013)], and it is possible that their prenylation and trafficking are regulated by signaling pathways that promote or suppress phosphorylation of their PBR.
• *What are the identities of the GEFs that regulate the prenylation and trafficking of small GTPases, and how do they interact with SmgGDS?*
Most studies of GEFs for Ras and Rho family members have focused on GEFs that activate prenylated small GTPases at membranes (Vigil et al., 2010; Gray et al., 2020). Very little is known about GEFs that interact with pre-prenylated small GTPases, or GEFs that interact with small GTPases as they complete the prenylation pathway and move to specific intracellular sites. The finding that the prenylation of some small GTPases is inhibited by the dominant negative mutation that suppresses GDP/GTP exchange (Berg et al., 2010) indicates that specific GEFs promote GDP/GTP exchange by pre-prenylated GTPases and facilitate their prenylation. The identification of these GEFs will provide important insights into the mechanisms that control the prenylation and trafficking of small GTPases.
• *How does SmgGDS participate in different diseases, and what are the best approaches to target SmgGDS therapeutically?*
It is well known that SmgGDS promotes cancer, and it is beginning to be recognized that SmgGDS also contributes to other pathologies, including neurological deficits (Asiri et al., 2020), and vascular abnormalities (Wang et al., 2017; Nogi et al., 2018; Renard, 2018). More studies are needed to define the roles of SmgGDS in these disorders and in other pathological conditions that involve abnormal activity of small GTPases. The therapeutic potential of SmgGDS SSOs (Brandt et al., 2020) and peptide inhibitors (Liao et al., 2020) is evident from recent pre-clinical cancer studies. However, with the crystal structure of SmgGDS now solved (Shimizu et al., 2018), developing small chemical inhibitors to disrupt interactions between SmgGDS and specific GTPase partners is a promising strategy to diminish the activity of oncogenic small GTPases in cancer, and potentially to regulate the activities of small GTPases that interact with SmgGDS in other disorders.
